# Olive Oil Phenolic Compounds’ Activity against Age-Associated Cognitive Decline: Clinical and Experimental Evidence

**DOI:** 10.3390/antiox12071472

**Published:** 2023-07-22

**Authors:** Anna Boronat, Gabriele Serreli, Jose Rodríguez-Morató, Monica Deiana, Rafael de la Torre

**Affiliations:** 1 Integrative Pharmacology and Systems Neurosciences Research Group, Hospital del Mar Research Institute, 08003 Barcelona, Spain; aboronat@imim.es (A.B.); jose.rodriguez@upf.edu (J.R.-M.); 2 Department of Medicine and Life Sciences, Universitat Pompeu Fabra, 08003 Barcelona, Spain; 3 Department of Biomedical Sciences, University of Cagliari, Cittadella Universitaria SS 554, 09042 Monserrato, Italy; gabriele.serreli@unica.it (G.S.); mdeiana@unica.it (M.D.); 4 Physiopathology of Obesity and Nutrition Networking Biomedical Research Centre (CIBEROBN), 28029 Madrid, Spain

**Keywords:** olive oil, extra virgin olive oil, phenolic bioactive compounds, cognitive decline, hydroxytyrosol, tyrosol, cognitive performance, neuroprotection

## Abstract

Epidemiological studies have shown that consuming olive oil rich in phenolic bioactive compounds is associated with a lower risk of neurodegenerative diseases and better cognitive performance in aged populations. Since oxidative stress is a common hallmark of age-related cognitive decline, incorporating exogenous antioxidants could have beneficial effects on brain aging. In this review, we firstly summarize and critically discuss the current preclinical evidence and the potential neuroprotective mechanisms. Existing studies indicate that olive oil phenolic compounds can modulate and counteract oxidative stress and neuroinflammation, two relevant pathways linked to the onset and progression of neurodegenerative processes. Secondly, we summarize the current clinical evidence. In contrast to preclinical studies, there is no direct evidence in humans of the bioactivity of olive oil phenolic compounds. Instead, we have summarized current findings regarding nutritional interventions supplemented with olive oil on cognition. A growing body of research indicates that high consumption of olive oil phenolic compounds is associated with better preservation of cognitive performance, conferring an additional benefit, independent of the dietary pattern. In conclusion, the consumption of olive oil rich in phenolic bioactive compounds has potential neuroprotective effects. Further research is needed to understand the underlying mechanisms and potential clinical applications.

## 1. Introduction

Recent estimates of the global prevalence of dementia and mid-term forecasts predict that the number of people affected by dementia worldwide will increase from 57.4 million in 2019 to 152.8 million cases in 2050 [1]. Given their increasing incidence, along with the lack of an effective cure and the limited success of pharmacological interventions, nutrition and dietary components have become increasingly important in preventing cognitive decline. In this regard, the Mediterranean Diet (MedDiet) has been identified as a potential preventive approach to reduce the risk of dementia. In fact, recent WHO guidelines recommended the MedDiet to reduce the risk of cognitive decline or dementia [2].

The traditional MedDiet is one of the most extensively studied dietary patterns in the world. Researchers have shown significant interest in it due to its numerous beneficial health effects [3]. Nowadays, and thanks to an exponential number of studies conducted over the last few decades, it is well-known that a high adherence to the traditional MedDiet confers significant protection against overall mortality, cardiovascular diseases, type 2 diabetes, obesity, and certain cancers [4,5]. From a dietary perspective, one of the main characteristics of traditional MedDiet is the use of extra virgin olive oil (EVOO) [6,7,8]. It is well-accepted that a significant part of the beneficial health effects attributed to the MedDiet is associated with EVOO consumption as the primary source of dietary fat and its phenolic compounds, some of which are known to be potent bioactive compounds [9]. Nonetheless, the exact mechanisms by which the MedDiet exerts protective effects in the brain have yet to be elucidated. However, it is well established that some MedDiet key food components exert antioxidant activity apart from anti-inflammatory, antiatherogenic, and pro-cognitive effects. These are relevant biological activities that could mitigate the onset and progression of neurodegenerative diseases.

The typical aging process involves brain alterations, affecting cognitive performance and adaptive functionality in the long run. Accumulated oxidative stress is a central facilitator of age-associated neurodegenerative diseases. Oxidative distress promotes cellular changes that eventually trigger significant dysfunction, increasing amyloid beta (Aβ) production and DNA damage, which further contribute to neurodegeneration and to the disease progression [10]. Based on this, the intake of exogenous antioxidants, like those present in EVOO, could have a positive impact on cognition and modulate brain aging and its manifestations through direct and indirect pathways [11].

Traditionally, the beneficial health effects of EVOO consumption were attributed to its high monounsaturated fatty acid (MUFA) content, primarily oleic acid (55–83%) in the form of triacylglycerides [11]. However, evidence has since emerged that the phenolic fraction, a minor part representing 1–2% of the total weight, contains bioactive compounds at a variable range (50–1000 mg/kg) that prevent oxidative damage [12] and provide benefits for plasma lipid levels following a dose-dependent relationship [13]. The EVOO phenolic compounds that have attracted the most scientific interest due to their beneficial effects are the phenolic alcohols hydroxytyrosol (HT) and tyrosol (Tyr) and their secoiridoids: oleuropein, ligstroside, oleacein, and oleocanthal [14]. In addition to determining EVOO’s organoleptic qualities, they have shown antioxidant, anti-inflammatory, cardioprotective, antiatherogenic, immunomodulatory, and anticancer activities [12]. Nonetheless, the actual capacity of a bioactive compound to exert significant effects on an aging brain is limited by several factors as its dosage, bioavailability, and the ability to cross the blood–brain barrier. Understanding the disposition of EVOO bioactive compounds is central for (i) comprehending the potential extrapolation of preclinical data (in vitro and animal models) to the clinical setting, and (ii) designing preclinical studies that are compatible with realistic physiologically achievable concentrations of these compounds in vivo.

The metabolic disposition of HT, Tyr, and their secoiridoids has been reviewed in detail elsewhere [14,15]. In brief, Tyr and HT absorption is matrix-dependent and takes place by bidirectional passive diffusion in a dose-dependent manner. While their absorption is reasonably good, their bioavailability is extremely poor due to extensive first-pass metabolism (phase I and II) in the gut and liver [15]. In phase I reactions, it is worth mentioning that Tyr, which has weaker antioxidant activity but is present in relevant concentrations in wine and olive oil, is biotransformed via CYP2D6 and CYP2A6 into HT [16]. The most abundant metabolites in plasma are the phase II sulphated and glucuronidated forms [17]. Several studies evidenced that sulphated and glucuronidated forms of Tyr and HT exert relevant biological activities [18]. Studies in animal models reported that both the unmodified forms and the metabolites of these compounds distribute in the organism in a concentration-dependent way, and can be found in given organs, such as the liver, the kidneys, and the brain [19]. Therefore, confirming their ability to cross the blood–brain barrier and to potentially exert neuroprotective activities [20]. 

In this article, we review the scientific evidence of the neuroprotective role of olive oil phenolic compounds in the prevention of age-associated cognitive decline. Our aim in the first part of the article was to review the mechanisms described in pre-clinical studies that could explain the results observed in epidemiological studies. We focused our revision around HT, which we considered to be the most relevant among the EVOO phenolic compounds. Acknowledging the presence of other relevant phenolic compounds like Tyr or oleuropein, it is important to note that they undergo conversion/metabolism into HT after its ingestion in vivo. Thus, HT plays a central role in the biological effects attributed to EVOO consumption. We focus our interest on those studies using doses compatible with those achieved in the diet and put special emphasis on the role of the metabolites of the phenolic compounds. In the second part, we critically review the main existing evidence from clinical trials assessing cognitive performance and the intake of olive oil phenolic compounds, either in the form of EVOO or in nutraceutical formulations.

## 2. Hydroxytyrosol Safety and Common Doses of Dietary Origin and in Functional Foods

The European Food Safety Authority (EFSA) released a health claim in 2011 regarding the ability of olive oil phenolic compounds to protect blood lipids from oxidation. Protective effects require a minimum daily intake of 5 mg of HT (per 20 g of olive oil) and its derivates [21]. This health claim was mainly based on results from EUROLIVE, a European Union (EU) FP5-funded project [13], and refers to phenolic compounds of dietary origin from olive oil. The estimated intake of HT in the EU adult population falls within the range of 0.13–6.82 mg/day/person [22]. Additionally, the ingestion of olives and red wine has been proposed as a contributor to the total HT exposure, resulting in higher daily ranges of HT intake (0.15 and 30 mg/day) [23].

There have been several requests to the EFSA Panel on Dietetic Products, Nutrition and Allergies (NDA) to deliver an opinion on HT, which is chemically synthesized, as a novel food (NF) pursuant to Regulation (EC) No 258/97 or to the Food and Drug Administration for its use as an antioxidant food additive or for the preparation of functional foods. The EFSA and the Spanish Agency for Consumer Affairs, Food Safety and Nutrition (AECOSAN) consider HT safe under the proposed uses and use levels [24,25]. The FDA in the United States considers HT to be a safe ingredient in processed foods at levels of 5 mg per serving, being the maximal exposure of 51.06 mg/person/day [26]. To reach these conclusions, the agencies considered the no observed adverse effect level (NOAEL) of 50 mg/kg body weight per day (from a sub-chronic oral toxicity study with the NF), the maximum anticipated daily intake for the NF, and the margin of exposure (MoE). It has also been considered that the expected daily intake of the NF would fall within the range of, or even be lower than, the exposure to HT from the consumption of olive oils and olives.

## 3. Considerations for Translating Pre-Clinical Studies to Human Subjects

In the evaluation of in vitro and in vivo studies conducted on animal models, it is essential to consider various factors, including the route of administration and doses, to accurately assess the translatability of observations to human subjects. For instance, by using a common equation allowing the transformation of doses in animal models to human equivalent doses (HED) [27].

HED (mg/kg) = Animal dose (mg/kg) × (Weightanimal [kg]/Weighthuman [kg]) ^(1–0.67)^

We prepared Table 1 for HT, comparing the doses used in rat and mice models with the corresponding HED and the total daily exposure in humans. In the table, we highlighted the doses ranging within the expected HT exposure in humans in bold. We have to consider that doses close to 50 mg are quite unlikely in subjects following the MedDiet and most probably doses in the range of 15 mg are more common in the general population consuming EVOO, olives, and red wine.

The route of administration in pre-clinical studies is also key to translating the doses and their effects to studies in humans. Oral administration is subject to factors such as the bioavailability, absorption, and first-pass metabolism, resulting in lower amounts of the compounds reaching the circulation. Conversely, intravenous and intraperitoneal administration routes result in higher deliveries of compounds into the bloodstream, but may not accurately mimic dietary intake. Several studies have shown that metabolites may contribute to biological activities, and the chemical species evaluated in preclinical models may not always be the most appropriate [28].

## 4. Pre-Clinical Evidence of the Role of Hydroxytyrosol and Derivatives in Neuroprotection

### 4.1. In Vitro Studies

Numerous in vitro studies have been conducted to shed light on the mechanisms underlying the potential of phenolic compounds in EVOO to prevent age-related cognitive decline. Considering the ample scientific evidence that ascertained the antioxidant and anti-inflammatory capacity of HT and its derivatives, most in vitro studies performed in recent years have investigated how this activity may translate into neuroprotection (Table 2).

#### 4.1.1. General Neuroprotection Models

An interesting study conducted by López de Las Hazas (2018) showed how HT and its acetated, sulphated, and acetated/sulphated metabolites were able to exert antioxidant and cytoprotective effects in vitro in neuroblastoma SH-SY5Y and neuronal-like LUHMES cells at concentrations similar to those achievable with the diet (2.5–10 μM) [20]. Differences in efficacy between metabolites and the parent compound were found, with the latter being more effective. Antiradical and neuroprotective activity of HT at two concentrations, the higher being non-compatible with diet (10–50 μM), was also tested by Omar (2017) again in SH-SY5Y cells treated with copper [29].

**Table 2:** Effects of HT and derivatives in in vitro neurodegenerative and Parkinson’s disease models.

Study	In Vitro Model	Compounds Tested	Concentrations	Significant Outcomes	Ref.
Yu et al., 2016	SH-SY5Y cells treated with 6-OHDA	HT	20–90 µM	Induction of the expression of phase II detoxifying enzymes NQO1, GST, GCL, and HO-1.	[30]
Crespo et al., 2017	Astrocytic cell line C6 exposed to Aβ (25–35)	HT	5 µM	Prevention of viability decrease through increased Akt activation.	[31]
Omar et al., 2017	SH-SY5Y cells treated with copper and H_2_O_2_	HT	10–50 μM	Antiradical and protective activity against peroxidation.	[29]
Funakohi-Tago et al., 2018	SH-SY5Y cells treated with 6-OHDA	HT, HT acetate and HT butyrate	5–10 µM	Reduction in the 6-OHDA-induced generation of ROS, activation of caspase-3, and subsequent cell death by HT butyrate, but not HT or HT acetate. HT butyrate induced Nrf2 and HO-1 expression	[32]
Hornedo-Ortega et al., 2018	Rat pheochromocytoma PC12 cells	HT	25–200 µM	Inhibition of α-synuclein fibrils formation and of their pro-inflammatory activity.	[33]
Lopez de Las Hazas et al., 2018	Neuroblastoma SH-SY5Y and neuronal-like LUHMES cells	HT, HT acetate, HT sulphate, HT acetate-sulphate	2.5–10 μM	Neuroprotection after oxidative injury observed after the pre-incubation with HT acetate.	[20]
Gallardo-Fernández et al., 2019	Murine microglial BV2 cells	HT	1–50 µM	Inhibition of α-synuclein aggregation and of NF-ĸB activation.	[34]
Leri et al., 2019	SH-SY5Y cells treated with Aβ1-42 oligomers	HT and oleuropein	0–20 µM	HT in synergy with oleuropein activated the autophagic flux to prevent cell damage. HT alone accelerated the formation of harmless fibrils while reducing harmful ones.	[35]
Hsu et al., 2021	Human cortical neuronal HCN-2 cells treated with rotenone	HT	30 μM	Inhibition of rotenone-induced cytotoxic responses by limiting Ca^2+^ entry. HT reversed ROS levels, cytotoxic responses, and antioxidant enzyme activities (SOD, GPX, and CAT) in rotenone-treated cells.	[36]
Mursaleen et al., 2021	hCMEC/D3-SH-SY5Y cell co-culture treated with rotenone	HT delivered through nanoformulations	20–200 µM	Encapsulation increased HT-induced protection against rotenone cytotoxicity and oxidative stress.	[37]
Visioli et al., 2022	7PA2 cell line transfected with cDNA encoding human amyloid precursor protein APP751	HT	5 µM	Increase of new mitochondria at 8 h post-HT treatment and increased mitochondrial fusion and ATP concentrations after 24 h of treatment with HT vs. untreated cells.	[38]
Nardi et al., 2023	SH-SY5Y cells treated with 6-OHDA	HT and derivatives esterified and encapsulated in nanoformulations	0.005–0.1 μM	Antioxidant capacity of the compounds tested. Better efficacy was observed after encapsulation.	[39]
Rivero-Pino et al., 2023	Human peripheral blood mononuclear cells treated with Aβ1-42 oligomers	HT	41 µM	Down-regulation of pro-inflammatory cytokine gene expression and of neutrophil activation.	[40]

6-hydroxydopamine (6-OHDA), amyloid β1-42 (Aβ1-42), catalase (CAT), glutamate–cysteine ligase (GCL), glutathione peroxidase (GPX), glutathione-S-transferase (GST), heme-oxygenase-1 (HO-1), NAD(P)H quinone dehydrogenase 1 (NQO1), nuclear factor erythroid 2–related factor 2 (Nrf2), nuclear factor ĸB (NF-ĸB), reactive oxygen species (ROS), superoxide dismutase (SOD).

#### 4.1.2. Parkinson Models

The prooxidant neurotoxin 6-hydroxydopamine (6-OHDA) is a compound used to simulate a condition similar to Parkinson’s disease (PD) in SH-SY5Y human neuroblastoma cells. The acetate and butyrate derivatives of HT were tested together with their free form to evaluate the protection of neuronal cells against 6-OHDA-induced apoptosis. Pre-treatment of SH-SY5Y cells with HT butyrate, but not with HT or HT acetate, significantly reduced 6-OHDA-induced reactive oxygen species (ROS) generation, caspase-3 activation, and subsequent apoptotic cell death. HT butyrate also induced Nrf2 protein expression. Its transcriptional activation resulted in the upregulated expression of heme oxygenase-1 (HO-1). The authors postulated that esterification with butyric acid increased HT lipophilicity and thus resulted in more efficacious effects due to its increased cell permeability [32]. In another study on the same experimental model, HT effectively induced the expression of phase II detoxifying enzymes, in addition to HO-1, namely NQO1, GST, and GCL, thus counteracting the deleterious effects exerted by dopamine and 6-OHDA [30]. However, it is noteworthy to signal that the concentrations used in the study were high (20–90 µM) and could not be comparable with those achievable in vivo through diet [41]. In a very recent study, HT-oleate and its derivative esterified with oleic acid were delivered at low concentrations (0.005–0.1 μM) with solid lipid nanoparticles in SH-SY5Y cells to counter 6-OHDA toxic effects. Results showed that the treatment inhibited the release of ROS, resulting in greater effectiveness than the treatment with non-encapsulated compounds [39]. Recently, two subsequent studies showed that HT restored neuronal functions in cells treated with the neurotoxic pesticide rotenone, used as an experimental model to induce neurotoxicity. In human cortical neuronal HCN-2 cells, HT (30 μM) prevented rotenone-induced Ca^2+^ signaling, cytotoxicity, and oxidative stress, improving antioxidant enzyme activities [36]. Additionally, HT was tested against rotenone in an hCMEC/D3-SH-SY5Y cell co-culture system to simulate the blood–brain barrier (BBB). HT (20–200 µM) was delivered through a nanoformulation that elicited substantial protective effects with respect to HT alone [37].

#### 4.1.3. Neurodegeneration Models

The accumulation of harmful protein aggregates in the brain is a defining characteristic of numerous neurodegenerative diseases and is believed to contribute to the cognitive decline observed in these conditions. Consequently, this feature has been the subject of many in vitro studies studying the neuroprotective activities of EVOO phenolic compounds (Table 2). Thus, the accumulation of protein aggregates was simulated by treating SH-SY5Y cells [35,42] and neutrophils [40] with Aβ1-42 oligomers to reproduce conditions similar to those encountered in Alzheimer’s disease (AD). In SH-SY5Y, HT in synergy with oleuropein, activated the autophagic flux in order to prevent cell damage by Aβ1–42 oligomers, while HT alone (0–20 µM) accelerated the formation of harmless fibrils to the detriment of harmful ones. In neutrophils, HT (41 µM) inhibited the proinflammatory effects of Aβ1–42 oligomers, limiting cell activation and thus contrasting neuroinflammation. The astrocytic cell line C6 exposed to Aβ (25–35) as a surrogate of AD, was incubated with HT (5 µM). After treatment with Aβ25–35, astrocyte viability significantly decreased compared to controls. Nonetheless, pre- and post-treatment with HT prevented this effect which was mediated by an increased Akt activation, a kinase involved in the insulin signaling pathway [31]. Similar studies performed against α-synuclein aggregation and its deleterious effects observed that both physiological and supraphysiological concentrations of HT (1–50 µM) reduced the induced inflammation by inhibiting nuclear factor ĸB (NF-ĸB) activation, the master regulator of the inflammatory response pathways in murine microglial BV2 cells [34].

The same research group has shown that HT, albeit at very high concentrations (25–200 µM), inhibits the formation of α-synuclein fibrils and their pro-inflammatory activity in rat pheochromocytoma PC12 cells [33]. Another work worth mentioning, conducted by Visioli (2022), assessed the effects of HT (5 μM) on mitochondrial energetic dysfunction in a cellular model of Aβ toxicity with a well-characterized mitochondrial dysfunction typically observed in AD. An increase of new mitochondria was observed at 8 h post-HT treatment, followed by higher mitochondrial fusion and increased ATP concentrations after 24 h of treatment with HT with respect to the untreated cells [38].

### 4.2. Animal In Vivo Studies

In the last decade, there has been an increase in the number of animal studies evaluating the neuroprotective effects of EVOO phenolic compounds, with a focus on the possible modulation of the distinctive features of brain degeneration.

#### 4.2.1. Capacity to Cross the BBB

Animal studies have provided evidence that EVOO phenolic compounds are capable of crossing the BBB, allowing them to potentially exert their beneficial biological activities in the brain. Following the administration of a nutritionally relevant dose of HT, its metabolites were detected in the brain tissue [19]. HT specifically was measured in rat brains after 5 min of intravenous injection (1.5 mg/kg); the brain tissue contained 0.31% of the administrated dose [43]. An interesting study described that HT preferentially accumulated in the hippocampus [44], a key site for spatial learning, memory, and emotional management, and one of the brain regions most damaged in the development of depression [45]. Additionally, it was demonstrated that the uptake of HT in the brains of rats was much higher in a pathological state, due to the increased BBB permeability [44], a common feature in several neurological diseases [46].

#### 4.2.2. Modulation of Oxidation/Inflammatory Pathways

Based on the in vitro capacity of olive oil phenolic compounds to modulate oxidative stress and inflammation, these pathways have been widely studied in animal models to elucidate the biological mechanisms of EVOO phenols to protect the aging brain (Table 3). These two pathways are key in the pathogenesis of neurodegenerative diseases such as AD and PD.

In a transgenic amyloid precursor protein/presenilin-1 (APP/PS1) mice model, HT (5 mg/kg/day) modulated mitochondrial oxidative dysfunction, as indicated by the reduction in mitochondrial carbonyl proteins and GSSG, the increased superoxide dismutase (SOD) expression, and the restoration of phase II enzymes’ expression [47]. HT intervention also reduced the levels of brain pro-inflammatory factors, which are induced in AD mice through the modulation of MAPK signaling pathways [47,48]. In a depressive mice model, HT (50 mg/kg) ameliorated oxidative stress in the hippocampus by enhancing SOD activity and reducing ROS and malondialdehyde (MDA) levels. HT also exerted anti-inflammatory effects, inhibiting tumor necrosis factor α (TNFα) and interleukin (IL)-1β expression, and upregulated the levels of brain-derived neurotrophic factors [49,50]. Lipopolysaccharides (LPS) are widely used to induce a strong pro-inflammatory response. HT treatment (100 mg/kg) in mice subjected to systemic injection of LPS strongly inhibited the increased expression of proinflammatory mediators induced in the brain, hence modulating neuroinflammation [51]. Improvements in cognition were observed in APP/PS1 AD mice fed with HT acetate (50 mg/kg/day) by the ameliorating of electrophysiological dysfunction of neurons and brain inflammation through the modulation of NF-κB activity and mitogen-activated protein kinase (MAPK) signaling [52]. HT-acetate effectively protected neurons from inflammation and apoptosis in AD mice by enhancing the transcription of mitochondrial ERβ, whose expression in frontal cortex neurons is significantly decreased in AD patients [52]. HT also improved mitochondrial function and prevented oxidative stress by activating the silent information regulator 1 (SIRT1)–AMP-activated protein kinase (AMPK) peroxisome proliferator-activated receptor gamma coactivator 1 (PGC1) axis in the brain of db/db mice, a model of type 2 diabetes [53], whose unregulated glucose metabolism is related to AD onset [54]. 

Monoamine oxidase (MAO) is an enzyme involved in breaking down neurotransmitters, and it is implicated in the pathogenesis of neurodegenerative diseases. Increased MAO activity is linked to decreased dopamine levels in PD and is thought to contribute to the formation of amyloid beta plaques in AD. MAO has also been shown to generate ROS, triggering oxidative stress and contributing to neurodegeneration [55]. In vivo, evidence of the neuroprotective effect of HT has recently been provided also in an 1-methyl-4-phenylpyridinium+ (MPP^+^) model of PD in rats. A single intravenous administration of 1.5 mg/kg of HT before intrastriatal infusion of MPP^+^ led to a significant reduction in the number of ipsilateral rotations, correlated with the preservation of striatal dopamine levels, due to the inhibitory effect of HT on MAO isoforms activity [56,57]. In the same model, pre-treatment of rats with HT and its derivatives, HT acetate, and nitro-HT, protected them from dopamine neuron degeneration, restoring MPP^+^-induced redox unbalance, as shown by the decrease of lipid peroxidation products and the rise of glutathione/glutathione disulphide ratio, giving further evidence of the main role of HT antioxidant action in neuroprotection [57]. A selective MAO-B inhibitory ability of HT was reported in a PD mice model, where HT (50 mg/kg) restored dopamine levels in the brain, prevented loss of dopaminergic neurons in the substantia nigra and striatum and improved behavioral deficits [58]. 

#### 4.2.3. HT in the Prevention of Neuronal Loss

Neurodegeneration is a complex process characterized by the progressive loss of neuronal function and structure. In vivo evidence of the ability of HT to improve neurogenesis, counteracting its physiological decline during aging, has been provided in a mice model of accelerated neural aging (Table 3). HT treatment (100 mg/kg/day) activated neurogenesis in the dentate gyrus, increased the survival of new neurons, and decreased apoptosis [59]. Studies in AD mice also highlighted the ability of HT to enhance the survival of neurons inhibiting cerebral cortex apoptotic responses by suppressing the mitochondria-mediated apoptotic pathway [47,48]. In a mouse model, HT (45 mg/kg/day) facilitated recovery after ischemic stroke by ameliorating stroke-associated learning and motor impairments. This was achieved through an increase in cerebral blood flow, functional and structural connectivity, and anti-inflammatory and neurogenic activity [60]. Notably, Arunsundar et al. also demonstrated that HT restored the expression of genes involved in the regulation of survival and memory functions: SIRT1, cAMP response element-binding protein (CREB), and CREB target genes that regulate cognitive functions and ADAM10, a SIRT1-regulated metalloprotease down-regulated in AD [48].

#### 4.2.4. Modulation of Cognition

The ability of HT to enhance cognitive performance in various animal models of age-related diseases and neurological dysfunctions associated with metabolic and genetic illnesses have been reported (Table 3). In a study performed by Arunsundar et al., HT treatment (10 mg/kg/day) reversed the deficit of spatial and working memory induced by intracerebroventricular injection of Aβ1–42 oligomer [48]. Similarly, in another mice transgenic model of AD, HT (5 mg/kg/day) improved electroencephalography activity and cognitive behavior [47]. In these two studies, HT amelioration of AD-related cognition impairment appeared independent of APP processing, as HT feeding did not attenuate brain Aβ accumulation. A significant improvement in cognitive function was observed in an AD genetic mice model supplemented with HT, with a 50 mg/kg dosage. In contrast to the previous studies, in these experimental conditions, behavioral and memory improvements were paralleled by a remarkable reduction in Aβ42 and pE3-Aβ deposits in the cortex and hippocampus [61]. Other studies in the invertebrate organism Caenorhabditis elegans models of PD showed the ability of HT to reduce neurodegeneration, increasing locomotion in worms suffering from α-synuclein-expression in muscles or rotenone exposure and preventing α-synuclein accumulation in dopaminergic and muscles cells [62,63].

Experiments based on in vitro and animal models have demonstrated that olive oil phenolic compounds such as HT and its metabolites are capable of protecting the brain from most of the molecular alterations that characterize the onset and progression of AD and PD. This effect is achieved primarily through the modulation of oxidative stress and neuroinflammation, as well as the reduction in deposition and toxicity of the altered proteins involved. As a result, studies using animal models have observed relevant improvements in cognitive performance. However, it is worth noting that concentrations used in these studies were high, and the bioactive compounds were administered acutely to achieve a measurable effect. These conditions are far from the physiological concentrations that are reached through the diet, which are lower and more extended in time. While in vitro and pre-clinical studies provide valuable information about the potential efficacy of olive oil phenolic compounds, caution should be exercised when extrapolating these findings to real-world scenarios.

**Table 3:** Effects of HT and derivatives in animal models.

Study	Animal Model	Compounds Tested	Dose	Route of Administration	Significant Outcomes	Ref.
Arunsundar et al., 2015	C57BL/6 mice treated with Aβ1–42 plus oA42i	HT	10 mg/kg/day for two weeks	Oral gavage	Reduction in brain pro-inflammatory factors (IL-18, IL-6, and COX-2) and modulation of MAPK signaling. Restoration of Bcl-2/Bad levels and activation of caspase-dependent mitochondria-mediated apoptotic pathway involving cytochrome c, APAF-1, and caspase-9/3.	[48]
Zheng et al., 2015	Specific pathogen-free female Sprague–Dawley rats exposed to restraint stress	HT	10–50 mg/kg/day for two weeks before mating	Oral	Prevention of stress-induced downregulation of neural proteins BDNF, GAP43, synaptophysin, NMDAR1, NMDANR2A, and NMDANR2B. Increase of transcription factors FOXO1 and FOXO3, and phase II enzyme-related proteins Nrf2 and HO-1.	[53]
Peng et al., 2016	Transgenic APP/PS1 mice	HT	5 mg/kg/day for six months	Oral gavage	Modulation of mitochondrial oxidative dysfunction, measured as reduction in mitochondrial carbonyl proteins and GSSG, increased SOD expression, and restoration of phase II enzymes. Restoration of p38 and JNK/MAPK signaling and attenuation of inflammation in the cerebral cortex. Inhibition of brain apoptotic responses.	[47]
Nardiello et al., 2018	TgCRND8 and wild type mice	HT	50 mg/kg for four weeks	Oral gavage	Reduction in Aβ42 and pE3-Aβ deposits in the cortex and hippocampus. Reduction in TNF-α expression, astrocyte reaction, and modulation of MAPKs signaling.	[61]
Calahorra et al., 2019	Male C57BL/6JRj mice which underwent transient occlusion of the right middle cerebral artery	HT	45 mg/kg/day for five weeks	Oral (Incorporated into the pellets)	Improved recovery after ischemic stroke by ameliorating stroke-associated learning and motor impairments. Increase in cerebral blood flow, functional and structural connectivity, and anti-inflammatory and neurogenic activity.	[60]
Brunetti et al., 2020	Wild type *C. elegans* strain N2 (Var. Bristol) and transgenic *C. elegans* strain OW13	HT	30 μg/mL, 100 μg/mL, 250 μg/mL and 500 μg/mL,	Oral	Enhancement of locomotion in worms suffering from α-synuclein-expression in muscles or rotenone exposure, reduction in α-synuclein accumulation in muscles cells, and prevention of neurodegeneration in α-synuclein-containing dopaminergic neurons.	[62]
D’Andrea et al., 2020	Btg1 knockout and Bgt1 wildtype strains (C57BL/6 background) mice	HT	100 mg/kg/day for 13 days	Oral (in drinking water)	Activation of neurogenesis in the dentate gyrus, increase of new neurons survival, and decrease of neuronal apoptosis.	[59]
Di Rosa et al., 2020	Wild type *C. elegans* strain N2 (Var. Bristol) and transgenic *C. elegans* strain OW13	HT	100–500 μg/mL.	Oral	Reduction in neurodegeneration, increase of locomotion in worms suffering from α-synuclein-expression in muscles or rotenone exposure and prevention of α-synuclein accumulation.	[63]
Pérez-Barrón et al., 2020	Male Wistar rats PD model treated with MPP^+^	HT	Single dose 1.5 mg/kg	Intravenous	Reduction in ipsilateral rotations, correlated with the preservation of striatal dopamine levels, due to the inhibitory effect on MAO activity.	[56]
Zhang et al., 2020	Male C57BL/6 mice treated with LPS	HT	Single dose 100 mg/kg	Oral gavage	Reduction in some pro-inflammatory mediators (COX-2, iNOS, TNF-α, IL-1β) levels and microglia/astrocyte activation in the brain.	[51]
Pathania et al., 2021	Male C57BL/6 mice treated with MPTP	HT	50 mg/kg/day for 1 week before and after MPTP	Oral gavage	Restoration of brain dopamine levels and prevention dopaminergic neurons loss in the substantia nigra and striatum by MAO-B inhibition.	[58]
Pérez-Barrón et al., 2021	Male Wistar rats PD model treated with MPP^+^	HT, HT acetate and nitro-HT	Single dose 1.5 mg/kg	Intravenous	Protection from dopamine neuron degeneration, restoration of MPP^+^-induced redox unbalance, decrease of lipid peroxidation products and rise of GSH/GSSG ratio.	[57]
Qin et al., 2021	Transgenic APP/PS1 mice	HT acetate	50 mg/kg/day for twelve weeks	Oral gavage	Improved escape latency and distance, and the number of platform crossings of AD mice in the water maze test by ameliorating neuronal apoptosis and modulating NF-ĸB activity and MAPK signaling.	[52]

1-methyl-4-phenyl-1,2,3,6-tetrahydropyridine (MPTP), 1-methyl-4-phenylpyridinium (MPP^+^), amyloid precursor protein/presenilin-1 (APP/PS1), amyloid β42 (Aβ42), apoptotic protease activating factor-1 (APAF-1), brain-derived neurotrophic factor (BDNF), cAMP response element-binding protein (CREB), chronic unpredictable mild stress (CUMS), cyclooxygenase-2 (COX-2), forkhead box protein O1 and O3 (FOXO1, FOXO3), glial fibrillary acidic protein (GFAP), glutathione reduced (GSH), glutathione oxidized (GSSG), Growth Associated Protein 43 (GAP43), heme-oxygenase-1 (HO-1), hypothalamic-Pituitary-Adrenal (HPA), ibotenic acid (oA42i), inducible nitric oxide synthase (iNOS), interleuchin 1β, 6, 18 (IL-1β, IL-6, IL-18), Janus kinase/mitogen-activated protein kinase (JNK/MAPK), monoaminoxidase (MAO), N-methyl-D-aspartate receptor 1/2A/2B (NMDAR1, NMDANR2A, NMDANR2B), nuclear factor erythroid 2–related factor 2 (Nrf2), nuclear factor ĸB (NF-κB), pyroglutamate-modified Abeta (pE3-Aβ), superoxide dismutase (SOD), tropomyosin receptor kinase B (TrkB), tumor necrosis factor α (TNF-α).

## 5. Clinical Evidence of the Role of HT and Derivates in Cognitive Decline

### 5.1. Intervention Studies Using OO with High Phenolic Compounds (EVOO or Others) and Changes in Cognitive Performance

Although there is a growing interest in the potential link between phenolic compounds found in olive oil (OO) and cognitive health, there is limited clinical evidence demonstrating their effectiveness in preventing cognitive decline. Despite promising findings from in vitro, animal, and epidemiological studies, little evidence stems from randomized clinical trials (RCTs). This fact could be related to methodological challenges in conducting preventive RCTs involving bioactive dietary compounds, particularly in relation to cognitive disorders and age-related conditions. For instance, short-term preventive interventions may not provide sufficient opportunity to assess the effects of interventions on cognitive function. Additionally, large sample sizes are required to control for many potential biases affecting cognitive performance. In the particular case of OO phenolic compounds, due to the absence of clinical trials administering them exclusively, we decided to include clinical trials administering (extra virgin) OO as a reasonable approach to study the effects of OO phenols on cognitive function. Based on this assumption, we have summarized the evidence from human studies linking OO phenols and cognitive decline; for further details of the studies refer to Table 4.

### 5.2. Clinical Trials Performed in Mediterranean Countries

#### 5.2.1. PREDIMED Study

The PREDIMED study was a RCT conducted in Spain to investigate the effects of a MedDiet on cardiovascular diseases prevention. The study involved over 7400 participants at high risk of developing cardiovascular disease, randomly assigned to three intervention groups: a MedDiet supplemented with EVOO (1 L/week), a MedDiet supplemented with nuts (30 g/day), and a low-fat diet as the control group [76]. The primary end point was a major cardiovascular event; however, in two subsamples of the study, cognitive performance was assessed.

Initially, a baseline cross-sectional evaluation of the association between polyphenol intake and cognitive function was performed by Valls-Pedret et al. The evaluation involved 477 PREDIMED participants who underwent a cognitive assessment. Urinary polyphenols, used as an objective measure of polyphenol-rich food total intake, were associated with memory performance following a continuous dose-dependent association. Further analysis of participants’ dietary pattern identified an independent association between total OO consumption and immediate verbal memory, as well as an association between virgin OO consumption and delayed verbal memory [64]. Likewise, the Navarra-PREDIMED node performed parallel studies to evaluate the cognitive performance and cognitive status of participants reported in two different publications. The mean follow-up for both studies was 6.5 years of nutritional intervention. The primary outcome was cognitive performance, and the secondary outcome was the incidence of mild cognitive impairment (MCI) and dementia. The first study regarding global cognitive performance included a broad group of participants (*n* = 522). The cognitive evaluation included the mini-mental state examination (MMSE) and the clock drawing test (CDT). Multivariate regression analysis showed that participants allocated to the MedDiet + EVOO group had better scores in MMSE and CDT than the control group [65]. The second study involved a smaller subsample of 285 participants who followed a more extensive cognitive evaluation focused on specific cognitive domains (memory, visual-spatial abilities, language, and executive function). Participants allocated to the MedDiet + EVOO exhibited significantly better cognitive performance than the control group, with significantly higher results in MMSE, immediate and delayed verbal memory (measured with ROCF), semantic and phonemic fluency (measured with FAS), and attention (measured with digit forward test). Compared with the MedDiet + nuts group, the EVOO group also exhibited better results in visual and verbal memory domains [66]. At the end of the nutritional intervention, participants allocated to the MedDiet + EVOO had a lower incidence of MCI compared to the control group [65]. Although the results described in both studies are of great importance, it is noteworthy to bear in mind that cognitive explorations were not included at baseline since they were not planned in the protocol. Consequently, it was not possible to assess temporal changes in cognition.

The last paper worth mentioning in the context of the PREDIMED trial is a longitudinal study conducted in the Hospital Clinic Barcelona node [67], evaluating cognitive performance. In this case, the median follow-up of the nutritional intervention was 4.1 years (*n* = 447). Cognitive performance tests were clustered into three composite scores: memory, frontal, and global cognition. At the end of the intervention, participants allocated to the MedDiet + EVOO group showed significantly better performance in frontal and global cognition composites (adjusted scores for changes from baseline). Overall, all the research performed assessing cognition in the PREDIMED study enabled the assessment of the long-term effects of a nutritional intervention on a large population through an RCT, which allowed detection of a greater magnitude of the effect previously reported in shorter studies that reported small benefits or null results.

#### 5.2.2. Clinical Trial: Replacement of Vegetables Oils for EVOO in Cognition

Mazza et al., in 2018, published the results of an RCT aimed to investigate the effect on cognitive performance of replacing all vegetable oils for a lower amount of EVOO (20–30 g/day) in the context of a MedDiet [68]. The clinical intervention included 180 healthy participants (≥65 years old) in the 1-year trial. The nutritional intervention consisted of a MedDiet supplemented with EVOO, while the control intervention was MedDiet recommendations. The cognitive evaluation included the longitudinal measurements of the MMSE and ADAS-cog tests. After one year of intervention, ADAS-cog scores improved in both groups; however, the improvement in the MedDiet + EVOO group was significantly larger than in the control group. This observation suggests that EVOO may confer added protection to the MedDiet in the face of age-related cognitive decline.

#### 5.2.3. Management of Mild Cognitive Impairment Patients with EVOO Study (MICOIL)

The MICOIL study is a 1-year longitudinal double-blinded RCT (*n* = 50) conducted in Greece. The intervention involved administering two types of EVOO differing in their phenolic content (high phenolic (HP) vs. moderate phenolic (MP)) as an add-on to the MedDiet recommendations, compared to only the MedDiet recommendations in MCI patients [69]. The primary outcome was cognitive performance, which included global cognition measurements (MMSE and ADAS-cog) and the evaluation of specific cognitive domains usually affected in MCI patients. At the end of the study, both HP-EVOO and MP-EVOO groups significantly improved ADAS-Cog test results (compared to baseline), with these changes being significant compared to the control group. Additionally, the HP-EVOO group improved in the letter fluency test, and the MP-EVOO group improved in the MMSE evaluation. A sub-analysis of APOE-e4 carriers (*n* = 29) showed cognitive performance stability or improvement, supporting the idea of the protective effects of the phenolic fraction of EVOO, even in at-risk patients, despite the small sample size. A subset of participants (*n* = 43) underwent electroencephalographic (EEG) resting-state recordings before and after the intervention. The EEG analysis found that all three dietary interventions produced significant changes in brain activity in MCI patients. Specifically, EVOO interventions improved dynamic functional connectivity, with the changes being more prominent in the HP-EVOO group but also observed in the MP-EVOO group. Furthermore, the HP-EVOO intervention reduced the over-excitation of information flow in spontaneous brain activity altering the signal spectrum of EEG rhythms, which led to an increase in human brain flexibility [70].

Finally, and in the context of the MICOIL study, biomarkers related to AD pathophysiology, namely the fibrinolytic system, oxidative markers, AD hallmarks, and neuroprotective proteins, were measured. The aim of two additional reports was to evaluate and compare the levels of these specific biomarkers across the three stages of AD progression, including healthy individuals and AD patients, who served as a reference for comparison with the MCI participants included in the MICOIL intervention. The study also monitored the 1-year evolution of the biomarkers with and without EVOO. At the end of the MICOIL study, MCI participants treated with EVOO showed significant improvement in their biomarkers profile, including a shift in fibrinolytic factors (PAI-1 and tPA), AD biomarkers (p-tau), and oxidative stress levels. These biomarkers improved to levels observed in cognitively healthy individuals. Contrarily, MCI patients without EVOO showed biomarker profiles that evolved in the direction of the profiles exhibited by AD patients [71]. The second MICOIL sub-study with a similar design found that MCI patients who received EVOO treatment upregulated their protein levels of BMI1, a neuroprotective factor. The EVOO treatment also modulated the levels of biomarkers associated with oxidative stress (p53) and inflammation (IL-6 and TNF-a), as expected [72]. The MICOIL intervention with EVOO may benefit MCI patients, potentially slowing or halting AD progression, as suggested by these findings. Additionally, the outcomes of these studies indicate potential mechanisms by which EVOO could exert its preventive functions and regulate the manifestation of crucial AD-related factors. However, further investigation is needed to understand the precise role that EVOO could play.

#### 5.2.4. PREDIMED PLUS Study

The PREDIMED-PLUS RCT was a subsequent trial that built upon the findings of PREDIMED. The trial is a 6-year, multicenter primary prevention trial and still ongoing. The aim of this second trial is to assess the long-term effects of an energy-restricted MedDiet on mortality and cardiovascular disease compared to only dietary counselling. The intervention also included physical activity promotion and behavioral support. All participants received a monthly supply of 1 L of EVOO to encourage adherence to the MedDiet and to promote compliance with the study protocol [77]. As in the first study, the primary outcome is the occurrence of clinical cardiovascular events. Differentially, the protocol included the evaluation of cognition for all participants. In a subsample of four recruiting sites, a more in-depth evaluation of cognitive performance (the PREDIMED-Plus-Cognition sub-sample) was administered.

An observational cohort study (*n* = 6647) assessed the longitudinal associations between baseline adherence to pre-specified dietary patterns and 2-year cognitive performance [73]. To explore further, a sub-analysis evaluated the implication of baseline consumption of individual food components on final cognitive performance. The study found that baseline use of OO was significantly linked with better scores in the global function composite score. Specifically, working memory capacity improved (measured with digit span test). However, it is worth noting that, despite the large population included, the study has inherent limitations since it performed associations between baseline diet and the main outcome in the frame of a nutrition intervention study, in which volunteers likely changed their diet. Additionally, the study evaluated OO as a single food category without distinguishing between refined oil (low in phenolic compounds) and virgin/extra virgin (high in phenolic compounds), making it difficult to discriminate whether the observed beneficial effect was coming from the specific lipid profile of OO, the contribution of phenolic compounds, or a combination of both.

The PREDIMED-Plus-Cognition included 487 participants that underwent an extensive neurocognitive evaluation. Overall, results indicated that higher adherence to the energy-restricted MedDiet was associated with more significant improvements in memory [78]. However, the study did not delve into the specific contributions of different dietary components such as EVOO. Unlike the first trial, EVOO was complementary and did not have a central role in the intervention. Nonetheless, these findings enlarge the evidence of the association between dietary patterns featuring EVOO as the primary source of fat and the preservation of cognitive function among older individuals.

### 5.3. Clinical Trials Administering OO Performed in Non-Mediterranean Countries

Kaddoumi et al. (2022) conducted a proof-of-concept clinical trial involving 26 patients with MCI, to evaluate the impact of EVOO on BBB permeability and brain function compared to refined olive oil (ROO) with a null phenolic fraction. The trial had a 6-month duration and included cognitive function measurements and AD biomarkers as secondary outcomes. Results revealed that EVOO treatment significantly reduced BBB permeability and enhanced functional connectivity compared to ROO. Both treatments improved the clinical dementia rating (CDR) and the delayed verbal memory (measured with WMS-IV) while decreasing the Aβ40/Aβ42 and p-Tau/t-Tau ratios [74]. Although the study had a small sample size, it effectively highlights the distinctive health advantages offered by the EVOO phenolic fraction complementary to the effects of the MUFA-specific profile of OO already present in refined OO.

### 5.4. Clinical Trials Administering Nutraceuticals with OO Phenolic Compounds

A nutraceutical formulation containing oleuropein, S-acetyl glutathione, piperine, bacopa, and vitamins B6, B12, E, and D3 was administered in an RCT [75] to mild AD patients. The initial study design was a crossover RCT with two 6-month intervention periods. The study design suffered because of the COVID-19 pandemic, and participants only had time to undergo one of the two interventions. The number of participants included decreased to 18 from the 40 initially included. Despite the small sample size and relatively short treatment period, the active treatment triggered statistically significant changes in almost all cognitive outcome measures, including global cognition, memory, attention, and executive function. Caution is needed in interpreting results since the trial was not placebo controlled and participants were not blinded to the treatment. These facts may bias the observed results. Additionally, the described effects cannot be attributed solely to oleuropein but probably to a synergistic effect of the active compounds in the nutraceutical formulation. The authors agree that, given the particularities of the trial, this represents a proof-of-concept study. They claim to be planning a double-blind RCT with a larger sample size and including validated AD biomarkers.

Finally, and as an additional point, we have examined the ongoing research and identified two registered RCTs specifically targeting EVOO phenols and age-related cognitive decline. The first study, identified as GOLDEN (NCT04440020), aims to evaluate the effect of a beverage made from OO leaves on cognitive performance in 100 patients with MCI (https://clinicaltrials.gov/ct2/show/NCT04440020; accessed on 20 June 2023). The second ongoing study is EVOCAD (NCT04229186), a pilot study investigating the effects of “coratina” EVOO in patients with MCI and AD. In this registered RCT, 24 participants will be administered either EVOO or refined OO. Researchers aim to evaluate its effect on cognitive performance, brain imaging, beta-amyloid, and other degradation biomarkers (https://clinicaltrials.gov/ct2/show/NCT04229186; accessed on 20 June 2023). The study has passed its completion date, its status has not been verified in more than two years, and publications reporting results are pending. 

Nonetheless, we have identified several ongoing trials whose primary outcome is cognitive performance through administering a MedDiet intervention. Since EVOO is known as a major contributor to the health benefits of a MedDiet, special attention should be paid in the future to the results obtained in these studies. Findings from all these ongoing studies will help to further enlarge the existing clinical evidence of the health effects of the phenols present in EVOO on cognitive maintenance.

## 6. Conclusions

In vitro and in vivo studies in cell tissue cultures and animal model surrogates of neurodegenerative diseases suggest that EVOO phenolic compounds, and particularly HT, may protect against pathological cognitive decline. These studies show that EVOO phenols can modulate oxidative stress and neuroinflammation, two essential pathways linked to the onset and progression of neurodegenerative diseases. The translational nature of the observations remains to be confirmed in clinical intervention studies with the phenolic compounds of EVOO. The reason is that, although in some cases in vitro and in vivo animal model doses evaluated are compatible with the exposure in humans, the bioavailability is so poor that probably in a few instances the low concentrations reached in biological fluids will compare to those achieved/tested in these models.

Regarding clinical studies, there is no evidence of direct effects of EVOO phenols on cognition. Alternatively, EVOO, the primary source of EVOO phenols, is part of different nutritional interventions in the context of dietary patterns, like the MedDiet, that explored their effects on different cognitive domains (Table 5). Nevertheless, these studies suggest that supplementing diets with OO high in phenol amounts results in cardioprotective and neuroprotective effects. Although the effect size is small, it is dose-dependent and quite reproducible. Further research is needed to understand the underlying mechanisms operating in neuroprotective effects.

## Figures and Tables

**Table 1:** Dose conversion factors of HT doses between rats and humans.

Humans		Mice	Rats
HED (mg/kg)	Total Dose (mg) ^a^	Dose (mg/kg) ^b^	Dose (mg/kg) ^c^
10	600	140.4	72.2
7	420	98.3	50.6
1.5	90	21.1	10.8
**0.75**	**45**	**10.5**	**5.4**
**0.25**	**15**	**3.5**	**1.8**
**0.01**	**0.6**	**0.14**	**0.07**

^a^ Total dose is calculated considering a standard adult of 60 kg of body weight. Reference weight was considered to be 0.02 kg for mice ^b^ and 0.15 kg for rats ^c^. Bold corresponds to the total doses achievable with the diet.

**Table 4:** Characteristics of the reviewed studies on the effects of olive oil and its cognitive effects.

Study	Type of Study	Intervention	Control Group	Health Status at Baseline	N and Duration	Measures of Cognition	Significant Outcomes of the Interventions	Ref.
Valls-Pedret et al., 2012	Cross-sectional	Not applicable	Not applicable	High cardiovascular risk	477 NA	Cognitive performance (MMSE, RAVLT, WMS, WAIS, and the Color Trail Test).	Total olive oil intake associated with immediate verbal memory.	[64]
							Virgin olive oil intake associated with delayed verbal memory.	
							Total urinary polyphenols associated with better immediate verbal memory.	
Martínez-Lapiscina et al., 2013	RCT	Int G1: MedDiet + EVOO (1 L/week)	Low fat diet	High cardiovascular risk	522 6.5 years	Cognitive performance (MMSE and CDT) and dementia/MCI incidence.	EVOO vs. control: better MMSE and CDT.	[65]
		Int G2: MedDiet + nuts (30 g/day)					EVOO: low odds ratio of MCI.	
Martínez-Lapiscina et al., 2013	RCT	Int G1: MedDiet + EVOO (1 L/week)	Low fat diet	High cardiovascular risk	285 6.5 years	Cognitive performance (MMSE, CDT, WMS, RAVLT, ROCF, BNT, FAS, WAIS-IIIR, and CDR).	EVOO vs. control: higher MMSE, ROCF immediate and delayed, FAS, and digital forward scores.	[66]
		Int G2: MedDiet + nuts (30 g/day)					EVOO vs. nuts: higher ROCF immediate and delayed and verbal (VPA) memory.	
Valls-Pedret et al., 2015	RCT	Int G1: MedDiet + EVOO (1 L/week)	Low fat diet	High cardiovascular risk	477 4.1 years (mean)	Cognitive performance (MMSE, RAVLT, ASF, DST from WAIS, color trait test, and WMS) in 3 composites: memory, frontal, and global cognition.	MedDiet + EVOO improved frontal cognition and global cognition adjusted composites for changes from baseline. Changes were significant compared to control group.	[67]
		Int G2: MedDiet + nuts (30 g/day)						
Mazza et al., 2018	RCT	MedDiet + EVOO (20–30 g/day)	MedDiet	Healthy ≥ 65	180 1 year	Cognitive performance (MMSE and ADAS-cog).	ADAS-Cog score showed greater improvement with MedDiet + EVOO vs. MedDiet.	[68]
Tsolaki M et al., 2020	RCT	Int G1: High phenolic (HP)- EVOO (50 mL/day)	MedDiet	MCI (60–80 years)	50 1 year	Cognitive performance (MMSE, RBMT, ROCF, Trail Making Test parts A and B, ADAS-Cog, WMS DST, fluency, and CDT).	HP-EVOO improved ADAS-Cog and letter Fluency (follow-up vs. baseline) compared to control group.	[69]
		Int G2: Moderate phenolic (MP)- EVOO (50 mL/day)					MP-EVOO improved MMSE and ADAS-Cog (follow-up vs. baseline) compared to control group.	
Dimitriadis S et al., 2021	RCT	Int G1: High phenolic (HP)- EVOO (50 mL/day)	MedDiet	MCI (60–80 years)	43 1 year	EEG resting-state with open eyes and close eyes conditions.	HP-EVOO decrease signal spectrum within 1–13 Hz and theta/beta.	[70]
							HP and MP-EVOOs improved the flexibility index being more noticeable in the HP- EVOO group.	
		Int G1: High phenolic (HP)- EVOO (50 mL/day)					HP-EVOO had a significant higher post-intervention reduction in non-linearity index.	
Tzekaki E et al., 2021	RCT/ observational	EVOO	MedDiet	3 groups: MCI, AD and healthy	84 1 year	Fibrinolytic system (levels of PAI-1, a2-antiplasmin, tPA).	EVOO reduced PAI-1, and tPA in MCI, restoring levels to those of healthy individuals.	[71]
						AD hallmarks (levels of p-tau, Aβ1-42, Aβ1-40).	EVOO reduced p-tau in MCI, restoring levels to those of healthy individuals and maintained AB-40 levels, downregulated in MCI without EVOO.	
						Oxidative stress: levels of MDA.	EVOO reduced MDA in MCI and restoring levels of healthy individuals	
Tzekaki E et al., 2021	RCT/ observational	EVOO	MedDiet	Three groups: MCI, AD and healthy	80 1 year	Levels of BMI1, p53, tau, p-tau, Aβ1–42, Aβ1–40, TNF-a, IL-6, and MDA.	EVOO increased BMI and decreases p53 and MDA in MCI patients.	[72]
							IL6 and TNF-a were downregulated in MCI patients by EVOO intervention.	
							12-month EVOO restored AD-related biomarkers (p-tau, Aβ1–42 and Aβ1–42/Aβ-40 ratio) to normal levels in MCI.	
Nishi et al., 2021	Observational	Not applicable	Not applicable	Overweight/obese + Metabolic syndrome	6647 2 year	Cognitive performance (MMSE, CDT, VFT-a and VFT-p, TMT A and B, DST-f and DST-b, and WAIS-III), and a global composite.	Baseline OO used as the primary oil was positively associated with changes in global cognitive function and in working memory (forward and backward DSTs).	[73]
Kaddoumi et al., 2022	RCT	EVOO (1200 mg/kg of total polyphenols) (30 mL/day)	Refined OO (null polyphenol content) (30 mL/day)	MCI (55–75 years)	26 6 months	MRI: contrast-enhanced MRI and fMRI.	EVOO decrease BBB permeability and brain connectivity.	[74]
						Cognitive performance (MMSE, CDR, WMS-IV).	EVOO and ROO decreased CDR and increased WMS-IV sub-sections.	
						AD biomarkers Aβ40, Aβ42, Tau, and p-tau181.	EVOO and ROO reduced Aβ42/Aβ40 ratio and p-tau/tau.	
Marianetti et al. in 2022	RCT	Nutraceutical formulation with SAG (50 mg), oleuropein (80 mg), vitamin B6 (1 mg), B12 (3 µg), vitamin E (15 IU), vitamin D3 (4 µg), piperine (3 mg), bacopa dry extract (100 mg) twice a day.	Absence of nutraceutical formulation	Mild AD	18 6 months	Cognitive performance (MMSE, CDT, RAVLT, RCF C, MA (attentive matrices), AAT, FAB, STEP, SVF, PVF).	Nutraceutical improved MMSE and CDT significantly vs. control group.	[75]
							Memory: nutraceutical improved RAVLT-immediate and delayed recall, and RCF-immediate recall vs. a deterioration in control group.	
							Attention: nutraceutical improved attentive matrices vs. a reduction was observed in control group.	
							Language and speech: nutraceutical improved AAT vs. no change in control group.	
							Executive functions: nutraceutical improved all measured indications vs. a decrease in control group.	

Alzheimer’s disease (AD); animals semantic fluency (ASF); attentive matrices (MA); clinical dementia rating (CDR); clock-drawing test (CDT); digit span test (DST) from Wechsler Adult Intelligence Scale (WAIS); extra virgin olive oil (EVOO); Frontal Assessment Battery (FAB); high phenolic extra virgin olive oil (HP-EVOO); mild cognitive impairment (MCI); mini-mental state examination (MMSE); moderate phenolic extra virgin olive oil (MP-EVOO); Progressive Matrices (Raven’s) (PVF); Rey Auditory Verbal Learning Test (RAVLT); Rey–Osterrieth complex figure copy (RCF C); randomized controlled trial (RCT); silhouettes subtest of the Visual Object and Space Perception battery (SVF); Stroop Test for executive processing (STEP); Wechsler Memory Scale (WMS); Wisconsin Card Sorting Test (AAT).

**Table 5:** Effects of EVOO phenolic compounds on the different cognitive domains.

Study	Intervention			Cognitive Domains			Ref
		Cognitive Deterioration	Memory	Attention	Fluency	Executive Function	
Valls-Pedret et al., 2012	Total OO	●	●	●	●	●	[69]
	Virgin OO	●	●	●	●	●	
Martínez-Lapiscina et al., 2013	EVOO	●	●	●	●	●	[70]
Martínez-Lapiscina et al., 2013	EVOO	●	●	●	●	●	[71]
Valls-Pedret et al., 2015	EVOO	●	●	●	●	●	[72]
Mazza et al., 2018	EVOO	●	●	●	●	●	[77]
Tsolaki et al., 2020	HP-EVOO	●	●	●	●	●	[73]
	MP-EVOO	●	●	●	●	●	
Nishi et al., 2021	Total OO	●	●	●	●	●	[4]
Kaddoumi et al., 2022	EVOO	●	●	●	●	●	[74]
Marianetti et al., 2022	Oleuropein	●	●	●	●	●	[75]

**Green:** significant results; **Grey:** not evaluated; **Yellow:** evaluated but no significant results were observed. Cognitive domains were classified as follows: cognitive deterioration (MMSE, CDT, ADAS-Cog), memory (RAVLT, ROCF and WMS-IV), attention (Digit forward test), fluency (FAS, animal and letter fluency) and executive function (ROCF, tests assessing working memory).
